# Spotlight on the microbes that produce heat shock protein 90-targeting antibiotics

**DOI:** 10.1098/rsob.120138

**Published:** 2012-12

**Authors:** Peter W. Piper, Stefan H. Millson

**Affiliations:** Department of Molecular Biology and Biotechnology, University of Sheffield, Firth Court, Western Bank, Sheffield S10 2TN, UK

**Keywords:** Hsp90, molecular chaperone, benzoquinone ansamycins, radicicol, drug resistance, fungal–plant interaction

## Abstract

Heat shock protein 90 (Hsp90) is a promising cancer drug target as a molecular chaperone critical for stabilization and activation of several of the oncoproteins that drive cancer progression. Its actions depend upon its essential ATPase, an activity fortuitously inhibited with a very high degree of selectivity by natural antibiotics: notably the actinomycete-derived benzoquinone ansamycins (e.g. geldanamycin) and certain fungal-derived resorcyclic acid lactones (e.g. radicicol). The molecular interactions made by these antibiotics when bound within the ADP/ATP-binding site of Hsp90 have served as templates for the development of several synthetic Hsp90 inhibitor drugs. Much attention now focuses on the clinical trials of these drugs. However, because microbes have evolved antibiotics to target Hsp90, it is probable that they often exploit Hsp90 inhibition when interacting with each other and with plants. Fungi known to produce Hsp90 inhibitors include mycoparasitic, as well as plant-pathogenic, endophytic and mycorrhizal species. The Hsp90 chaperone may, therefore, be a prominent target in establishing a number of mycoparasitic (interfungal), fungal pathogen–plant and symbiotic fungus–plant relationships. Furthermore the Hsp90 family proteins of the microbes that produce Hsp90 inhibitor antibiotics are able to reveal how drug resistance can arise by amino acid changes in the highly conserved ADP/ATP-binding site of Hsp90.

## 2. Introduction

Heat shock protein 90 (Hsp90) is essential for the conformational maturation, activation and maintenance of proteins needed for all of the six hallmarks of cancer: angiogenesis, immortalization, metastasis, impaired apoptosis, insensitivity to antigrowth signals and autocrine growth [1,2]. As a result, this promiscuous molecular chaperone is a prime target for drug development, with 13 Hsp90 inhibitor compounds now being in phase I/II clinical trials for the treatment of various cancers [1,2]. Not only does the continual growth of these cells in hostile micro-environments need sustained Hsp90 chaperone activity, but the Hsp90 in cancer cells has a higher affinity for Hsp90 inhibitor drugs than the Hsp90 in normal cells, a reflection of the high levels of Hsp90-dependent protein complexes in cancer cells [3]. It is probable that Hsp90 inhibitor drugs will, in due course, find applications in a number of other therapies. Because they are potent activators of the heat shock response, one of their actions is to elevate cellular levels of the molecular chaperones that assist protein folding. This, in turn, can help to restore losses of proteostasis and thus ameliorate the effects of those diseases that are caused by loss of proper protein conformation (e.g. cystic fibrosis, as well as neurodegenerative diseases of ageing [4–6]). In addition, Hsp90 inhibitors are of potential use for the treatment of infectious disease, because in eukaryotic cells Hsp90 is essential for the replication of both viruses (e.g. picornaviruses [7]) and pathogenic microbes (e.g. the malaria parasite *Plasmodium falciparum* [8]).

## 3. The discovery of natural product inhibitors of heat shock protein 90

**Figure 1.**
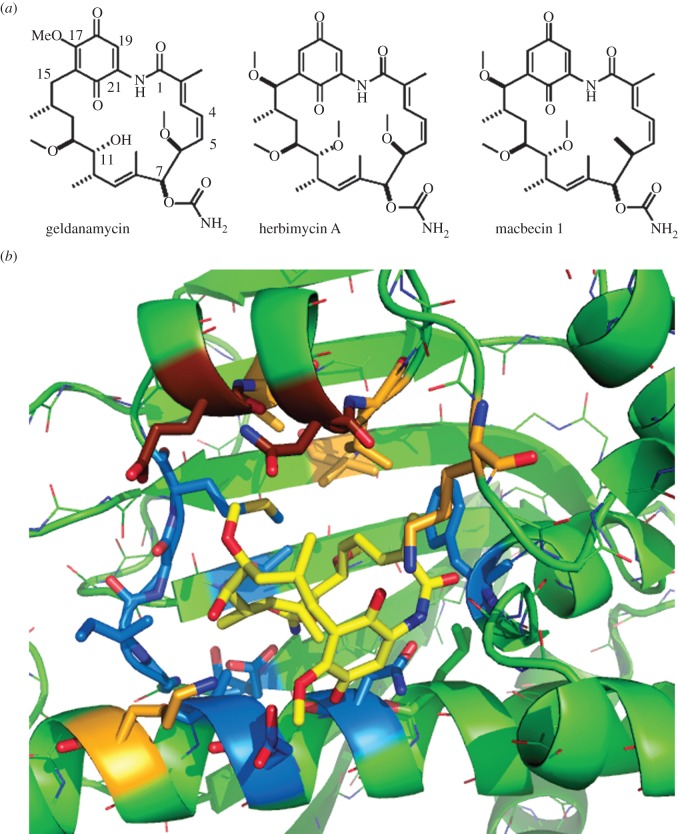
(*a*) Benzoquinone ansamycins studied as Hsp90 inhibitors: geldanamycin (GdA), herbimycin A and macbecin 1. (*b*) GdA, shown in yellow, in the conformationally constrained *trans-*conformation that it adopts when bound within the N-terminal domain of the yeast Hsp90 (PDB ID: 1A4H). Hsp90 residues forming its binding site that are conserved in the HtpG of the GdA-producing organism *S. hygroscopicus* are shown in blue, whereas those in this site that are altered in the *S. hygroscopicus* HtpG are shown in light or dark brown. The two polar residues indicated in dark brown are the ones which generated partial resistance to GdA in yeast cells when altered to *S. hygroscopicus* HtpG-specific residues in the native Hsp90 of yeast [21].

**Figure 2.**
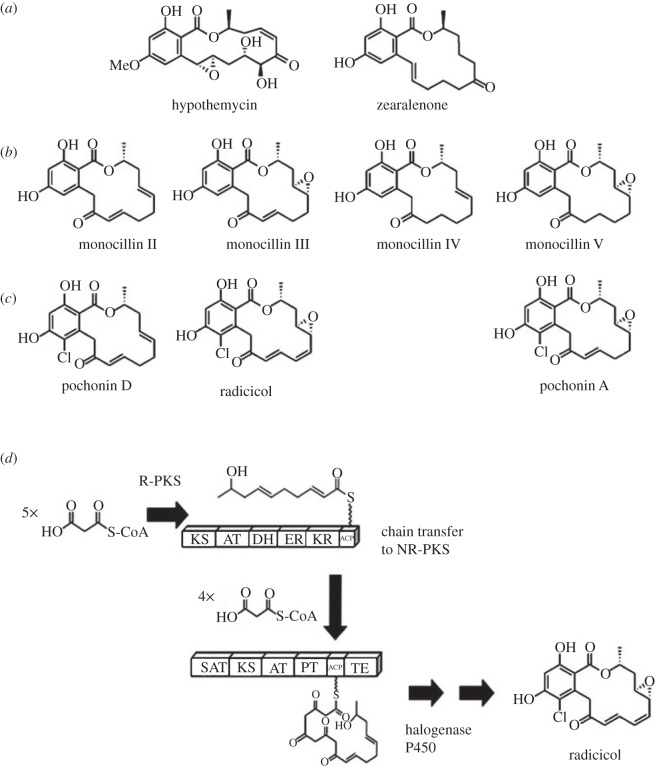
(*a*–*c*) Fungal-derived resorcyclic acid lactones: (*a*) hypothemycin and zearalenone; (*b*) the monocillins; (*c*) RAD, pochonin A and pochonin D, chlorinated forms of the monocillins that are inhibitors of Hsp90. (*d*) A simplified version of the RAD biosynthetic pathway, involving the sequential actions of a reducing polyketide synthase (R-PKS), a non-reducing PKS (NR-PKS), a halogenase and a P450 oxygenase [28].

The appreciation that Hsp90 might be a valuable drug target was initially slow in coming. It was initiated by studies on the actions of benzoquinone ansamycins (table 1), actinomycete-derived antibiotics of very closely related structure (figure 1), in mammalian cell culture. In 1970, geldanamycin (GdA) was reported as exerting potent activity against L1210 mouse leukaemia and KB cells [9]. Later a modified form of GdA (17-dimethylamino-geldanamycin) was found to be 20-fold more potent against *v*-src tyrosine kinase-transformed 3Y1 cells as compared with the untransformed parental 3Y1 cells, providing the first indication that cancer cells may be particularly susceptible to derivatives of GdA [22]. This, in turn, was followed by the landmark discoveries that an immobilized form of GdA binds Hsp90, and also that GdA and herbimycin A act to reverse the oncogenic transformation of fibroblasts by *v*-src tyrosine kinase by blocking the formation of the Hsp90–*v*-src heteroprotein complex [23]. GdA was subsequently shown to promote the degradation of a number of Hsp90-dependent (Hsp90 ‘client’) proteins *in vivo* [24], and to inhibit the ATPase activity of Hsp90 through binding, with very high degree of selectivity [25], within the ADP/ATP-binding site of the Hsp90 N-terminal domain [26,27]. A little later radicicol/monorden (RAD) (figure 2*a*; table 1), a non-ansamycin fungal-derived antibiotic that had previously also been found to revert the transformed phenotype of *v-*src-expressing fibroblasts [29], was found to bind within this same ADP/ATP-binding site on Hsp90, but by making substantially different bonding interactions within this chaperone site [30]. Several benzoquinone ansamycin derivatives [31], as well as a number of small molecule synthetic inhibitor compounds based on the interactions of RAD, are now in clinical trials [1,2].

**Table 1.** Natural product inhibitors of Hsp90.

sourced from microbes:	
benzoquinone ansamycins	antibiotics derived from actinomycetes; notably geldanamycin (GdA) from *Streptomyces hygroscopicus* var. *geldanus* [9]; herbimycin A from *S. hygroscopicus* strain AM-3672 [10]; and macbecin 1 from *Nocardia* sp. No C-14919 [11]
RAD and pochonins; (figure 2).	resorcyclic acid lactones produced by several fungi of the Sordariomycetes taxon; pochonins A and D from *Pochonia chlamydosporia* have been shown to directly inhibit Hsp90 [12]
novobiocin, coumermycin A1, clorobiocin	coumermycin family antibiotics from *Streptomyces spheroides*; bind the C-terminal region of Hsp90 [13]
sourced from plants:	
harmine	an alkaloid from the African medicinal plant *Guiera senegalensis*; displays a higher affinity for the N-terminal domain of Hsp90 from the malaria parasite *Plasmodium falciparum* than the corresponding domain of the human Hsp90 [14]
epigallocatechin-3-gallate	a naturally occurring polyphenol from the green tea, *Camellia sinensis*; binds the C-terminal domain of Hsp90 [15]
derrubone	a prenylated isoflavone isolated from the Indian tree *Derris robusta* [16]
gedunin and celastrol	triterpenes isolated from the Indian neem tree *Azadirachta indica* [17]; celastrol binds the Hsp90 C-terminal domain [18]
withaferin A	a steroidal lactone from the Indian medicinal plant *Withania somnifera*; binds C-terminal domain of Hsp90 [19]
gambogic acid	a compound from the plant species *Garcinia hanburyi*; binds the N-terminal domain of Hsp90 at a site distinct from the ADP/ATP-binding pocket [20]

Since these discoveries of Hsp90 as the target of the benzoquinone ansamycins and RAD, several other natural compounds—including a number derived from medicinal plants—have been found to bind, and inhibit, this chaperone (table 1). Each of these is now providing a lead for further drug development [20,32]. However, in this review, we focus solely on the benzoquinone ansamycins and the fungal-derived resorcyclic acid lactones (RALs), because these are the natural inhibitors where high-resolution crystal structures have revealed in atomic detail the interactions made as they bind within the Hsp90 N-terminal domain. Also RAD (an RAL; figure 2c) is still the most potent natural product inhibitor of Hsp90 discovered to date (binding to Hsp90 with a *K*d of 19 nM [30]). We suggest why the microbial species producing these secondary metabolites might exploit Hsp90 inhibition. We also discuss how the Hsp90 family proteins of these microbes can provide insights into how the extremely conserved ADP/ATP-binding pocket of Hsp90 can be altered to generate forms of Hsp90 with a degree of resistance to these antibiotics. Because the Hsp90 inhibitor drugs in cancer clinical trials all act by binding within an almost identical ADP/ATP pocket on the corresponding human Hsp90s, these microbial Hsp90s provide an indication of whether the Hsp90 in cancer cells may be able to mutate this site to drug resistance, yet still remain functional.

## 4. Why might *Streptomyces hygroscopicus* use heat shock protein 90 as an antibiotic target?

Streptomycetes are soil-dwelling mycelial bacteria forming sporulating aerial branches. *Streptomyces hygroscopicus*—the organism producing GdA and herbimycin A (figure 1)—belongs to the *Streptomyces violaceusniger* clade of these bacteria, a clade that is attracting interest both for its production of secondary metabolites and for its abilities to antagonize the growth of a number of plant-pathogenic fungi [33]. The *S. hygroscopicus* gene clusters directing the biosynthesis of GdA and herbimycin A are well characterized [34]. Synthesis of both compounds entails a chain extension from the basic ‘building block’ of the ansamycins, 3-amino-5-hydroxybenzoic acid, this chain extension and a subsequent cyclization producing the polyketide synthase (PKS)-derived carbon skeleton (progeldanamycin), upon which various post-PKS modification enzymes act to produce the differences in substitution patterns at carbon positions 11, 15, and 17 (figure 1*a*; for details see [34]). Though a full chemical synthesis of GdA has been achieved, it entailed 20 steps and a low overall yield [35]. The production of benzoquinone ansamycin-based drugs is therefore usually achieved by chemical modification of the GdA or herbimycin A derived from *S. hygroscopicus* fermentation. However, both the natural benzoquinone ansamycins (figure 1*a*), as well as their chemically-modified forms, have severe drawbacks in clinical use owing to their low solubility and the redox activity of the benzoquinone ring. The benzoquinone, through redox cycling and the production of the unstable semiquinone radical, is able to generate appreciable oxidative stress *in vivo* [36]. For this reason *S. hygroscopicus* has recently been genetically engineered for the production of new non-quinone analogues of GdA, compounds with a better pharmacological profile than the natural antibiotics [37]. Without the unravelling of the details of GdA and herbimycin A biosynthesis [34], this could not have been achieved.

Intuitively one suspects that streptomycetes must produce antibiotics so as to have a competitive advantage against the other micro-organisms that they encounter. However, because antibiotic production is usually delayed until most of the growth has been completed, its main purpose may be to defend the colony biomass against overgrowth by other organisms rather than help in the competition for primary biomass accumulation. While the extracellular biology of streptomycetes is extremely complex, it is known that these species often establish close interactions with fungal hyphae [38]. Furthermore, a number of potent inhibitors of fungal growth (e.g. hygromycin B, nigericin, rapamycin) are amongst the diverse range of antibiotics produced by different isolates of *S. hygroscopicus*. Potentially the Hsp90 of fungi may be an ideal target for yet another actinomycete-produced antibiotic, because this chaperone is critical for a number of the activities needed for biosynthesis of the cell wall and the response to cell wall stress in fungal cells [39,40]. The production of Hsp90 inhibitors by certain *S. hygroscopicus* may, therefore, help these streptomycetes inhibit the fungi in soil.

## 5. Why might fungi use heat shock protein 90 as an antibiotic target?

The fungal-derived Hsp90 inhibitors in table 1 are a subset of a diverse range of RALs produced by different fungal species. The biosynthesis of two of these RALs—hypothemycin and zearalenone (figure 2*a*)—is particularly well characterized, the synthesis of zearalenone having being studied in both *Gibberella* and *Fusarium* species (see [41] for literature). In essence, fungal RALs are initially built up from units of malonyl-CoA through the sequential actions of a reducing polyketide synthase (R-PKS) and a non-reducing PKS (NR-PKS; figure 2*d*), then subsequently acquiring much of their structural diversity through different post-PKS modification reactions. The pathway of RAD biosynthesis has been revealed with analysis of the RAD biosynthetic gene clusters of two species: *Chaetomium chiversii* [42] and *Pochonia chlamydosporia* [28,41]. Each gene cluster encodes an R-PKS, a NR-PKS and just two post-PKS tailoring enzymes, a cytochrome P450 and a non-haeme halogenase. The latter introduce the epoxide and the chlorine, respectively (figure 2*d*) [28].

Diverse fungi are currently identified as producers of the structurally similar RAD, the pochonins or the monocillins (figure 2*b,c*). They include mycopathogens, plant pathogens, as well as species forming endophytic or mycorrhizal symbioses with plants. As long ago as 1950, RAD and its unchlorinated monocillin precursor (figure 2*b*) were identified in *Monocillium nordinii*, a destructive mycoparasite of pine stem rusts in North America [43]. It was later suggested that these compounds might play a key role in the mycoparasitic actions of this *M. nordinii* [44]. Often remarkably high amounts of RAD, pochonins or monocillins have been found in fungal extracts. High RAD production was identified for *Cylindrocarpon radicicola*, a fungus isolated from the mycorrhizal tuberous roots of a saprophytic orchid [45]. Monocillin was present at approximately 30 per cent by weight in dry extracts of *Paraphaeosphaeria quadriseptata,* a fungus that inhabits the rhizosphere of cacti as well as young maize plants [46,47]. Similarly, up to 10 per cent RAD was identified in extracts of the above-mentioned *Chaetomium chiversii,* an endophytic fungus that colonizes plant stem tissue [48]. This efficient production of Hsp90 inhibitor compounds may reflect the Hsp90 chaperone being a prominent target in the establishment of certain parasitic (mycoparasitic, nematophagous), plant-pathogenic (fungal pathogen–plant) and symbiotic fungal–plant interactions.

Parasitic fungi may potentially have a very important ecological impact through their actions of reducing the populations of nematodes and fungal oospores in soil. They have, therefore, attracted attention as possible agents for the biocontrol of these plant pathogens. The RAD and pochonin-producer *Pochonia chlamydosporia*, mentioned above, infects and destroys the eggs of root knot and cyst nematodes [49]. Another RAD-producing species, the mycoparasitic fungus *Humicola fuscoatra*, negatively impacts on a number of the important oomycete plant-pathogenic fungi (e.g. species of the *Phytophthora* genus) by infecting their oospores in soil [50]. Extracts of this and other RAD- and monocillin-producing fungi have been under consideration for the biocontrol of *Aspergillus flavus* and *Fusarium verticillioides*, two mycotoxin-producing, seed-infecting fungal pathogens [50,51]. While the events whereby a mycoparasitic fungus invades oospores have been studied by microscopy [52], there appears to have been little detailed investigation of whether the production of an Hsp90 inhibitor by such a species might impact on this process.

Production of Hsp90 inhibitors by fungi might also reflect the importance of Hsp90 in the plant defence against pathogens. Pathogen entry to a host plant is accompanied by a local defence response. Instrumental in this response are the plant immune sensing NLR (*n*ucleotide-binding domain and *l*eucine-rich *r*epeat containing) proteins. NLR proteins are ‘clients’ of the Hsp90 chaperone [53,54], whose rapid degradation in Hsp90-deficient mutant plants results in a greater sensitivity to microbial pathogens [55]. Oomycete pathogens such as species of *Phytophthora* suppress this immune response by secreting into the plant cells effector proteins, the latter defined by an RXLR host cell entry motif [56]. With the additional production of an Hsp90 inhibitor able to target the Hsp90 needed for the stability of the plant's NLR proteins, this effector protein action should be potentiated. It has been suggested that the RAD and monocillin production displayed by the maize pathogen *Colletotrichum graminicola* may assist the fungus by disrupting the maize plant defences this way and by excluding other fungi from necrotic tissues [51].

As regards an endophytic fungal/plant or mycorrhizal fungal/plant symbiosis, the fungal production of an Hsp90 inhibitor may be perceived as facilitating the establishment and maintenance of the symbiotic relationship in two ways. First, it may lead to the selective inhibition of other fungi, including some potential plant pathogens. Second, by suppressing the activity of the plant Hsp90, it may compromise the plant immune (NLR protein) sensing system and lead to the induction of stress (heat shock) proteins [47]. Entry of a mycorrhizal fungus to the root system of a host plant is known to be accompanied by a local defence response, similar to the response to pathogens. Establishment of the symbiotic relationship may be facilitated if this defence response is suppressed.

## 6. What can the organisms that make heat shock protein 90 inhibitors tell us about heat shock protein 90 inhibitor resistance?

Resistance acquired through mutation of the drug target still remains one of the most serious causes of the failure of cancer chemotherapy. This is highlighted by the treatments with highly selective inhibitors of oncogenic tyrosine kinases, where many patients with advanced stage disease respond initially but then relapse. Analysis of clinical samples has shown that resistance to the Bcr-Abl kinase inhibitor imatinib generally arises either though Bcr-Abl gene amplification or with a single amino acid substitution in the Abl kinase domain [57]. The latter, a change to the ‘gatekeeper’ residue, a threonine that engages in a critical hydrogen bond with the drug molecule bound within the Abl kinase domain, is a ‘generic’ resistance mutation that threatens to reduce the potency of any ATP-competitive inhibitor designed against a tyrosine kinase. Mutation of this gatekeeper threonine is also the cause of resistance to drugs that target the mutated forms of the epidermal growth factor receptor, a situation where its primary effect is not to compromise drug binding, but to increase the affinity of the kinase domain for ATP [58].

The Hsp90 inhibitors in clinical trials present the distinct advantage that resistance would not arise this way. While these drugs are also competitive inhibitors of ATP binding, their mode of binding to their target is very different from that of the above protein kinase inhibitors. Hsp90 is a member of a protein superfamily with novel ATP-binding properties, a superfamily that includes such diverse proteins as DNA topoisomerase II, DNA mismatch repair enzymes MutL and histidine kinases. The singular unifying feature of this superfamily is the unconventional Bergerat ATP-binding fold, a site where ATP binds with its ribose ring in the C3′-endo conformation, rather than with the ribose in the more usual C2′-endo configuration as seen in the classical mode of ATP binding by kinase enzymes [27].

This ADP/ATP-binding pocket on Hsp90 is evolutionarily very highly conserved, being composed of essentially identical amino acid residues in practically all Hsp90 family proteins from eubacteria through to eukaryotes. A mutation causing drug resistance by weakening the binding of the drug within this site would involve changing one of these invariant residues. As these are the amino acids that facilitate the ATP binding or ATPase steps of the Hsp90 chaperone cycle, such a mutation would be expected to severely compromise, if not totally inactivate, Hsp90. We have been investigating the Hsp90 family proteins of microbes that make GdA or RAD on the premise that, should nature have been able to evolve a drug resistant form of Hsp90 altered in this site, it would most probably occur in these species so as to confer a degree of resistance to this antibiotic production.

A single Hsp90 family protein (HtpG) is encoded within the genome of *S. hygroscopicus* var. *geldanus*, the organism that makes GdA [21]. When purified following bacterial expression, this *S. hygroscopicus* HtpG was found to display normal binding affinities for nucleotides and RAD, but no detectable binding of benzoquinone ansamycins [21]. It, therefore, provides a blueprint for an Hsp90 that retains the binding of nucleotides and RAD, yet fails to bind GdA. Within this *S. hygroscopicus* HtpG, there are changes to several of the conserved amino acids that facilitate the interactions with GdA in the cocrystal structures of this antibiotic bound to eukaryotic forms of Hsp90 [26,30]. Most of these changes lie on one side of the drug-binding pocket (figure 1*b*). Unexpectedly, we found that these amino acid substitutions found in the *S. hygroscopicus* HtpG did not lead to loss of the essential Hsp90 function when introduced into the sole Hsp90 of a model eukaryotic cell (the yeast *Saccharomyces cerevisiae*). Therefore, the ADP/ATP-binding pocket of Hsp90, though highly conserved in evolution, is still amenable to mutational change. Furthermore, most of these amino acid changes did not generate GdA resistance when inserted into the Hsp90 of yeast. Only two of them generated partial resistance, these (the mutations E88G and N92L) involving replacement of polar amino acid side chains for uncharged side chains on a small α-helix that comprises one face of the ADP/ATP-binding pocket (figure 1*b*). Together these two mutations generated approximately a 10-fold weaker affinity for GdA *in vitro* and 2.5-fold increases in IC(50) for GdA and 17-allylaminodemethoxygeldanamycin inhibition of yeast growth *in vivo* [21]. Importantly, E88G and N92L were unique amongst the changes to the ADP/ATP site of *S. hygroscopicus* HtpG in that they alone generated in the yeast Hsp90 a greater relative increase in *K*_d_ for GdA, when compared with *K*_d_ for ADP *in vitro*, as would be required for greater ATP versus GdA occupancy of this nucleotide-binding site *in vivo*. A crystal structure revealed that they weaken the interactions of the chaperone with the C-12 methoxy group on GdA (figure 1) [21].

**Figure 3.**
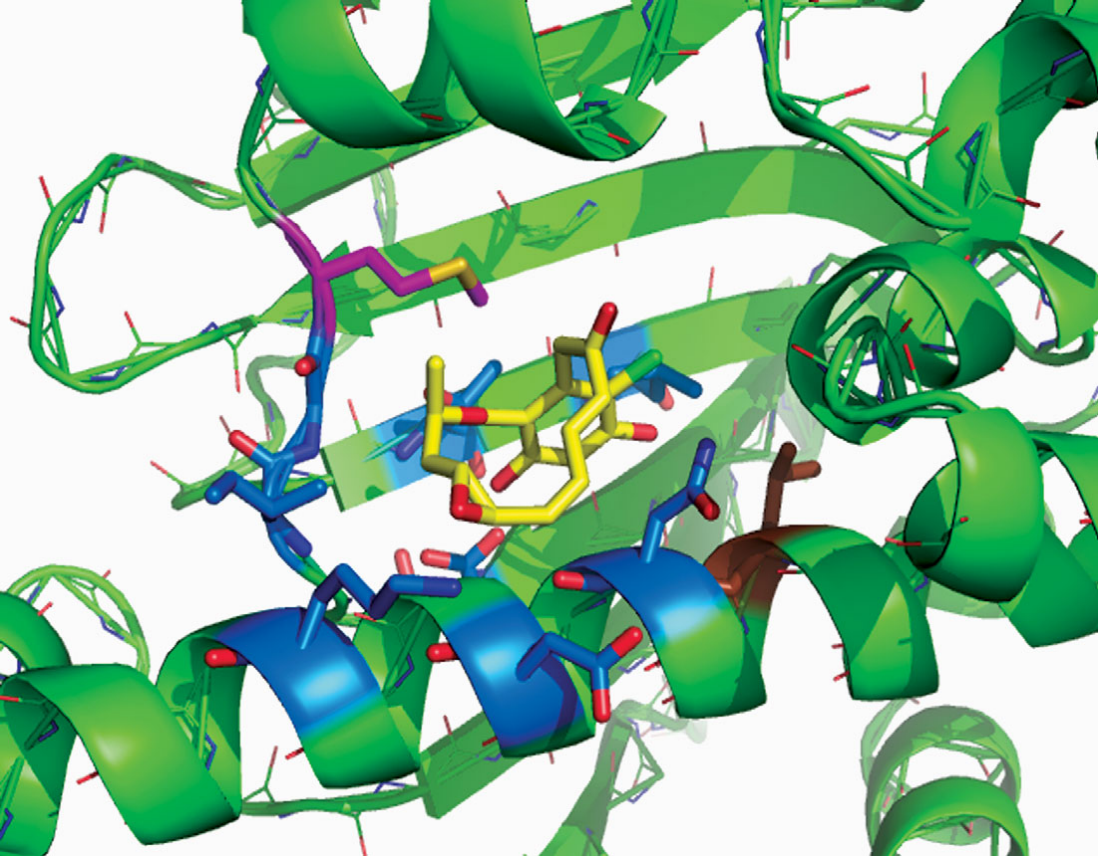
RAD, shown in yellow, bound within the N-terminal domain of the yeast Hsp90 (PDB ID: 1BGQ). Hsp90 residues forming the binding site of this antibiotic molecule are shown in blue. Indicated in brown is the conserved leucine residue that is altered to an isoleucine in the Hsp90 of *Humicola fuscoatra*, thus generating partial RAD resistance [60]. Indicated in magenta is the conserved methionine (Met84) that is altered to phenylalanine in Hsp90 of the maize pathogen *Colletotrichum graminicola*.

Most fungi have just a single, essential, cytosolic form of Hsp90 [59]. We investigated this Hsp90 from one of the fungi producing RAD (the mycoparasite *H. fuscoatra*) to see whether it is altered in its binding of ligands. The *H. fuscoatra* Hsp90 was found to have a normal affinity for nucleotides and GdA, but approximately eightfold weaker than normal binding affinity for RAD [60]. However, unlike the *S. hygroscopicus* HtpG, *H. fuscoatra* Hsp90 is largely unaltered in the conserved amino acid residues of the Hsp90 ADP/ATP-binding pocket. Its only novel feature is an isoleucine replacement of normally conserved leucine at the base of this pocket (figure 3). When introduced into the Hsp90 of yeast, this single, conservative leucine to isoleucine change reproduced the weakened binding of RAD displayed by the *H. fuscoatra* Hsp90 *in vitro* and, when expressed in the sole Hsp90 of yeast cells, generated partial resistance to RAD *in vivo* [60]. A crystal structure indicated that this leucine to isoleucine substitution acts to weaken RAD binding by causing an increase in hydration in the vicinity of the bound RAD molecule [60]. Remarkably, Hsp90 of the RAD- and monocillin-producing fungal pathogen of maize, *Colletotrichum graminicola*, is altered in yet another highly conserved residue, a methionine at the entrance to the ADP/ATP-binding pocket (figure 3). While the effects of this methionine to phenylalanine change have yet to be investigated, it is possible that it too weakens the binding of these polyketides.

## 7. Conclusions

The secondary metabolites of soil-dwelling actinomycetes and fungi have long been the source of new antibiotics and drug leads. The discovery of antibiotics highly selective for Hsp90, and the subsequent development of drugs based upon the molecular interactions made by these antibiotics within the ADP/ATP-binding site of Hsp90 are a classic example of this. Much attention now focuses on the clinical trials of these drugs, especially as these are the only cancer chemotherapeutic agents known to impact all six hallmarks of cancer simultaneously [1,2]. While promising activity has now been demonstrated in certain malignancies, certain of the other trials have proved disappointing [1,2]. Designing effective therapies around Hsp90 inhibitors will entail a proper understanding of the molecular mechanisms whereby cells are often refractory to their effects. Many aspects have to be considered: such as differences in drug action on the various isoforms or conformations of Hsp90, dissimilarities in drug metabolism, the effects of Hsp90 inhibition on both normal and tumour cell physiology, the ability to cause either cell stasis or apoptosis, or an inability to achieve an effective level of drug in the tumour [36]. Most importantly, these inhibitors cause a strong activation of the heat shock response, leading to increases in the cellular levels of stress proteins, proteins with largely pro-survival/anti-apoptotic functions [36]. Drugs based on other, more recently identified natural inhibitors of Hsp90 (table 1) may be able to overcome some of these problems. Thus the induction of the anti-apoptotic heat shock response seen with drugs that bind to the ADP/ATP site of the Hsp90 N-terminal domain may be avoided by switching to a new class of Hsp90 inhibitor based on the interactions of novobiocin, the latter an agent that interacts with the C-terminal region of the chaperone (table 1; [61]).

However, inhibitors binding within the N-terminal ADP/ATP site of Hsp90 have the potential attraction that resistance might not emerge readily by mutation within this site. To date, it would appear that study of the Hsp90 family proteins of organisms that make Hsp90-targeting antibiotics partly validates this prediction. Thus only partial, not complete, resistance to either GdA or RAD has been generated by introducing into the Hsp90 of yeast cells the unusual features of the ADP/ATP-binding site of the *S. hygroscopicus* HtpG or the *H. fuscoatra* Hsp90. Furthermore, the *S. hygroscopicus* HtpG—though resistant to GdA—still has normal affinities for RAD and for other inhibitors (NVP-AUY922 and VER49009) whose binding is based on the interactions of RAD [21]. Conversely, the leucine to isoleucine change in the *H. fuscoatra* Hsp90—while it generates partial resistance to RAD—does not affect the binding of GdA [60]. This suggests that, should mutations such as these within the ADP/ATP-binding site of human Hsp90 ever cause a degree of drug resistance in the clinic, it should be possible to overcome this resistance by switching from an Hsp90 inhibitor based on the interactions of GdA to one based on the interactions of RAD, or vice versa. This is not to say that mutations located elsewhere in Hsp90 cannot lead to resistance. Indeed an increased Hsp90 inhibitor resistance is apparent with mutations that increase the ATPase activity of the chaperone, at least in cell culture systems [62,63].

Microbes making Hsp90 inhibitors may be resistant to their antibiotic production for reasons other than a weakened binding of the antibiotic to the chaperone itself. While the *S. hygroscopicus* HtpG exhibits no detectable binding of GdA [21], one cannot state with confidence that this confers *S. hygroscopicus* with resistance to its production of this antibiotic. Not only is HtpG protein absent, therefore nonessential, in a number of prokaryotes [59], but there is still no evidence for any specific function for HtpG in a streptomycete. Furthermore, antibiotic resistance in *S. hygroscopicus* is sometimes due to enzymes that modify the antibiotic molecule [64]. Similarly, while the Hsp90 of the RAD-producing fungus *H. fuscoatra* has a low binding affinity for RAD [60], other factors may be of rather greater importance in rendering such a fungus resistant to this antibiotic. Plasma membrane drug efflux pumps elevate the cellular resistances of yeast to GdA and RAD, acting to lower the intracellular levels of these inhibitors [62]. Potentially such efflux activities could be elevated in a RAD-producing fungus. Furthermore, as with the streptomycetes, production of antibiotics by fungi such as *H. fuscoatra* may be a starvation response, occurring at locations distal from the actively growing hyphal tips and serving mainly to defend the hyphal biomass against overgrowth by other organisms. Alternatively RAD resistance might arise through reductions in the normal strong requirement for Hsp90 in fungal growth [39,40]. In those species where it has been investigated, the signalling circuitry of fungi is heavily dependent on Hsp90 [65]. Fungi that make Hsp90 inhibitors may be adapted to partially overcome this, such that a study of these species may be highly instructive in terms of understanding the global influences of the Hsp90 chaperone.
